# Symptoms and functional limitations related to respiratory health and carbon monoxide poisoning in Tanzania: a cross sectional study

**DOI:** 10.1186/s12940-022-00847-x

**Published:** 2022-04-02

**Authors:** Thomas Zoller, Elirehema H. Mfinanga, Tresphory B. Zumba, Peter J. Asilia, Edwin M. Mutabazi, David Wimmersberger, Francis Mhimbira, Frederick Haraka, Klaus Reither

**Affiliations:** 1 Swiss Tropical and Public Health Institute, Socinstr. 57, 4051 Basel, Switzerland; 2 Ifakara Health Institute, Dar es Salaam, Tanzania; 3 Department of Infectious Diseases and Respiratory Medicine, Charité – Universitätsmedizin Berlin, corporate member of Freie Universität Berlin, Humboldt-Universität zu Berlin, and Berlin Institute of Health, Berlin, Germany; 4 University of Basel, Basel, Switzerland

**Keywords:** Carboxyhaemoglobin, Respiratory symptoms, Tanzania

## Abstract

**Background:**

The burden of chronic respiratory symptoms and respiratory functional limitations is underestimated in Africa. Few data are available on carbon monoxide (CO) poisoning in sub-Saharan Africa and existing data is derived from CO in ambient air, but not from biomarkers in the blood.

**Methods:**

Data from the Tanzanian Lung Health study, a cross-sectional study on lung health among outpatients and visitors to an urban as well as a rural hospital in Tanzania, was analyzed to describe respiratory symptoms and functional limitations. Saturation of peripheral blood with carbon monoxide (SpCO) was measured transcutaneously and non-invasively in participants using a modified pulse oxymeter indicative of CO poisoning. Univariate and multivariate analysis was performed.

**Results:**

Nine hundred and ninety-seven participants were included in the analysis, the median age of participants was 46 years (49% male). 38% of participants reported some degree of chronic shortness of breath and 26% felt limited in their daily activities or at work by this symptom. The median SpCO was 7% (IQR 4–13, range 2–31%) among all participants without active smoking status (*N* = 808). Participants cooking with gas or electricity had the lowest SpCO (median 5%), followed by participants cooking with charcoal (median 7%). Cooking with wood, particularly using a stove, resulted in highest SpCO (median 11.5%). Participants from households where cooking takes place in a separate room had the lowest SpCO as compared to cooking outside or cooking in a shared room inside (6% vs. 9% vs.10.5%, *p* < 0.01). Sex or the activity of cooking itself was not associated with a difference in SpCO. Multivariate analysis confirmed cooking in a separate room (as compared to cooking outside) and living in a rural vs. urban setting as protective factors against high SpCO.

**Conclusion:**

The findings demonstrate a high burden of chronic respiratory symptoms which also cause socioeconomic impact. High levels of SpCO indicate a relevant burden of carbon monoxide poisoning in the local population. The level of CO in the blood is more dependent on shared exposure to sources of CO with the type of housing and type of cooking fuel as most relevant factors, and less on person-individual risk factors or activities.

**Supplementary Information:**

The online version contains supplementary material available at 10.1186/s12940-022-00847-x.

## Background

Urban as well as rural populations in Tanzania are frequently exposed to hazards with negative impact on lung health. Typical threats to lung health in Africa are exposure to dusts and fumes originating from cooking indoors or cooking on an open fire outdoors, exposure to dusts and fumes at work or cigarette smoking causing diseases like chronic bronchitis and chronic obstructive pulmonary disease (COPD) [1–6]. Symptoms like shortness of breath or chronic coughing can have major impact on daily life and even more importantly, on the ability to work and to generate income [6]. Recent data show that in Africa a substantial burden of COPD – ranking 3^rd^ in the causes of mortality worldwide – exists with major social and economic impacts [7–9].

Combustion of biomass fuels in households [10] and smoking [11] are major sources of carbon monoxide (CO) exposure in populations worldwide. In developing countries, many people use biomass fuels for cooking outdoors and even indoors resulting in a high burden of CO exposure [11]. A study from Malawi found evidence for chronic CO poisoning (mean carboxyhaemoglobin (CO-Hb) 5.8%) in children under 5 years old [12]. In non-smokers, CO-Hb levels of 1,5%-2% are considered normal and in smokers levels of > 10% can be found [13, 14]. Adverse cardiovascular effects can be expected above 2% and neurobehavioural effects ≥ 5% CO-Hb; detailed data on adverse health effects of CO poisoning are described by the U.S. Department of Health and Human Sciences [14]. From a pathophysiological point of view, chronic CO poisoning further reduces the oxygen (O2) transport capacity of the blood and may aggravate the widespread problem of anaemia in Africa [15]. Most studies from Africa published to date have measured personal exposure to CO [16, 17] or measurements of indoor and/or outdoor air [5, 18]; these methods are however limited by the remaining uncertainty regarding the relationship of CO concentration in the air versus the actual CO-Hb in the blood relevant for pathophysiologic effects.

This study aims to describe symptoms and functional limitations related to lung health in Tanzania. In contrast to previous studies measuring exposure in ambient air, the individual burden of carboxyhaemoglobin in the blood was determined as saturation of peripheral blood with CO (SpCO) transcutaneously and non-invasively in the blood of study participants in an urban as well as in a rural African real-world setting. Moreover, the study aims to demonstrate whether adverse health effects including negative social impact are associated with individual carbon monoxide levels in the blood.

## Methods

**Fig. 1:**
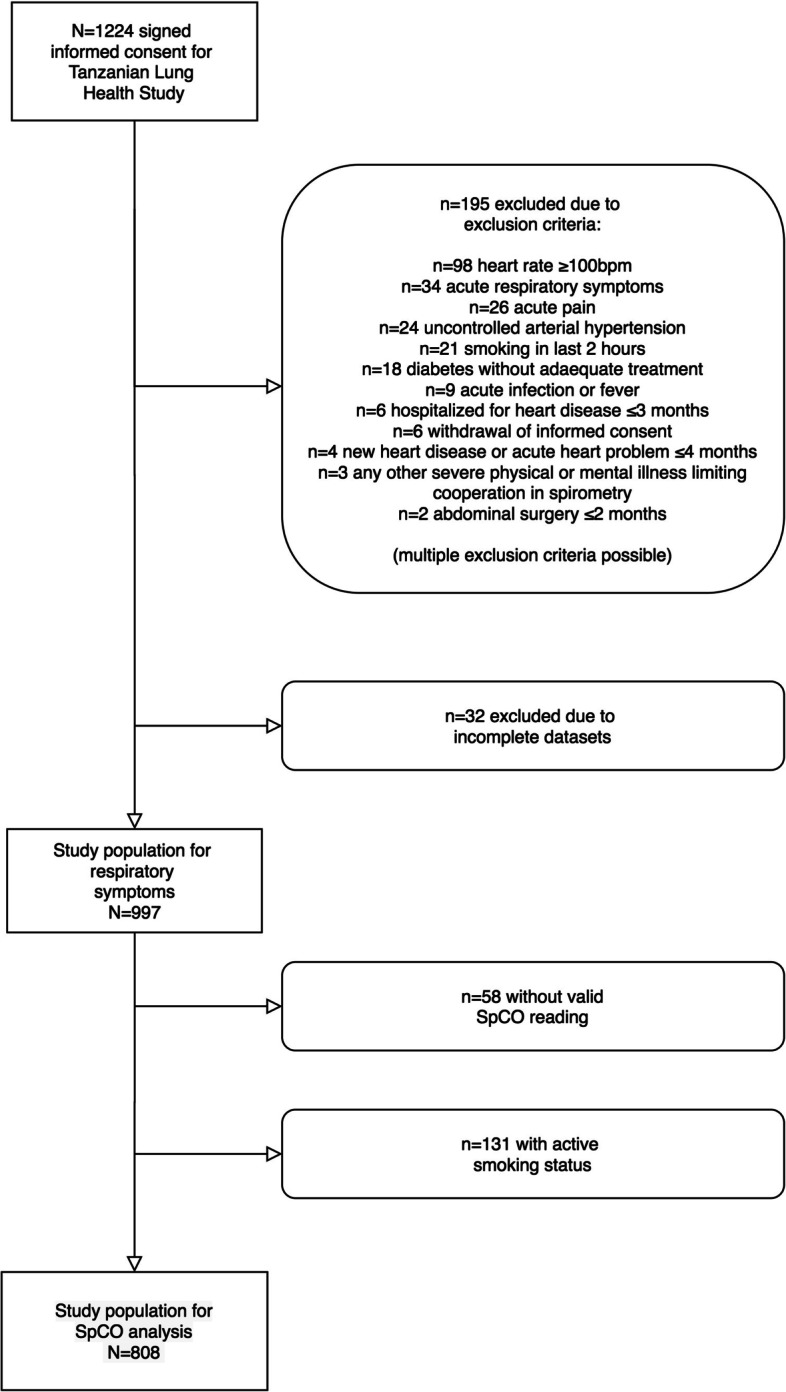
Overview of study populations

A cross-sectional facility-based study (the “Tanzanian Lung Health Study”, TLHS) was conducted among attendants of the outpatient department, persons accompanying patients and visitors to the rural Bagamoyo District Hospital (Bagamoyo, Pwani, Tanzania), and the urban Mwananyamala Regional Hospital (Dar es Salaam, Tanzania) between October 2015 and September 2016. The study collected baseline data on lung health as well as lung function data. Participants with a minimum age of 18 years were included if they were either (i) attendants of a primary healthcare clinic for any reason other than an acute respiratory problem or (ii) persons accompanying patients or visitors to the hospital visiting inpatients. Participants with contraindications to perform spirometry or other study-related procedures and patients presenting with acute respiratory symptoms were excluded (see Fig. 1 for full exclusion criteria). To allow analysis of outcomes in participants across all relevant age groups in this study, age-stratified sampling was used. Participants were invited randomly: depending on the number of persons available and waiting in the outpatient department, e.g. every 5th patient and, if available, an accompanying person was invited for participation if the age matched the required age stratum. In case participation was declined, the next waiting person was invited. In case no participants were available in the waiting area, visitors to the hospital visiting inpatients were invited for participation. Spirometric data on chronic pulmonary obstruction were reported previously, which also was the relevant parameter for sample size calculation [6]. A sample size of 600 participants was intended to provide an acceptable prevalence estimate with 2.5% precision for TLHS assuming a prevalence of 10% of chronic airflow obstruction. After obtaining informed consent either in writing or by an impartial witness, participants were interviewed by a trained physician or a study nurse in a separate room of the outpatient department to obtain data on lung health and TB history. Participant information was entered directly into a purpose-built (not validated) electronic questionnaire (supplementary Fig. 1) using OpenDataKit (www.opendatakit.org) software. Moreover, participants were trained in performing spirometry supported by a video. Between the interview and spirometry, height and weight was measured and all participants were examined with a pulse oxymeter capable of measuring peripheral fractional oxygen saturation (SpO2), fractional carbon monoxide saturation (SpCO) in per cent and heart rate per minute by analysis of absorption of light at different wave lengths in a fingertip sensor. A Masimo® Rad-57 pulse oxymeter (CE-marked) connected to a Rainbow® fingertip sensor was used. A single reading was taken using an index finger of the participant in sitting position after at least 3 min. The reading was considered valid if a stable and homogeneous pulse signal for at least 15 s was observed and, according to the manufacturer´s instructions, if the perfusion index given by the instrument was > 1%, and there was no low signal warning by the device. The manufacturer specifies the accuracy of the reading for S_PCO_ as ± 3% compared to an invasive blood gas analysis across all age groups.

The primary outcome of this analysis was the level of CO poisoning in participants of TLHS. Self-reported symptoms and limitations in work or other activities of daily life caused by respiratory conditions or linked to chronic CO poisoning were secondary outcomes.

Data were analysed using JMP ver. 14 and SPSS ver. 28. Descriptive statistics were used to characterize respiratory symptoms and limitations of participants. Some answers to questions (e.g. for distinction between biomass and non-biomass fuels were) regrouped. Levels of SpCO were compared between participants exposed / not exposed to risk factors using the Mann–Whitney-U-test to compare two and the Kruskal–Wallis-Test to compare multiple groups. X_2_ test was used to compare categorical outcomes between groups. For multivariate analysis, multiple linear regression as well as logistic regression models were used. Non normally distributed dependent variables were power transformed. Variables were included if they had a clinicial or physiological influence on the dependent variable and/or if they were significant in univariate analysis at *p* ≤ 0.1. The final models were fitted using stepwise variable selection for multiple linear regression and forward variable selection for logistic regression in SPSS (inclusion for F ≤ 0.05, exclusion for F ≥ 0.1). Details on the respective models used are given in the table legends. Differences were considered significant when *p* was < 0.05.

## Results

*N* = 1224 participants signed informed consent for TLHS, *N* = 997 participants were available for analysis of respiratory symptoms and *N* = 808 participants for analysis of effects of CO poisoning (for details see Fig. 1).

The median age of the study population was 46 years (IQR 35–57 years) across the different age groups invited for participation (for details see Table 1). There was an equal gender distribution.

**Table 1:** Demographic characteristics of study population in *N* = 997 participants

		n	% of study population
Sex	Male	490	49.1
	Female	507	50.9
Site	Bagamoyo	691	69.3
	Mwananyamala	306	30.7
Type of participant	Outpatient of hospital	414	41.5
	Accompanying person or visitor to hospital	583	58.5
Age group	18–29	170	17.1
	30–39	143	14.3
	40–49	289	29.0
	50–59	178	17.9
	60–69	125	12.5
	70–79	79	7.9
	> 80	13	1.3
Main occupations (category only listed if > 5% of respondents)	Business and trade	284	28.5
	Farming	246	24.7
	Not working	149	17.2
	Craftsmen	44	5.1
Reasons for seeking medical attention (among patients, category only listed if ≥ 10% of respondents)	Infections / fevers	72	17,4
	Musculosceletal problems and injuries	59	14.2
	Cardiovascular problems	54	13.0
	Gastrointestinal problems	45	10.9
	Chronic respiratory problems	51	12.3
Smoking status	Never smoked before	751	75.3
	Active smokers	131	13.1
	Former smokers (refrained ≥ 6 months)	115	11.5
Regular cooking	Yes	544	54.6
	No	406	40.7
Method of cooking	Charcoal	605	60.7
	Wood in an open fire	230	23.0
	Gas	76	7.6
	Electricity	3	0.3
Predominant methods of cooking in Bagamoyo (rural)	Charcoal		57
	Firewood		33
	Gas		6
Predominant methods of cooking in Mwananyamala (urban)	Charcoal		69
	Firewood		2
	Gas		12

Main occupations were business and trade (28.5%) and farming (24.7%) and the leading reasons for seeking medical attention for patients were infections/fevers as well as injuries and musculosceletal, cardiovascular, gastrointestinal, and chronic respiratory problems (for details see Table 1).

### Determinants of lung health in the study population

75.3% of participants had never smoked before and 13.1% were active smokers (Table 1). 11.5% were former smokers who had refrained from smoking at least for six months. Of those participants with a smoking history, 117 (47.6%) reported not more than one pack per year. Active and ex-smokers were predominantly male (88,5% and 80,9% respectively). 574 participants (57.7%) reported exposure to cigarette smoke at home or at the workplace (passive smoking).

54.6% of participants cooked regularly for the family or friends and 40.7% did not cook regularly. The majority of participants cooked with charcoal (60.7%) and others used wood in an open fire (23%), gas (7.6%) and electricity (0.3%). Regarding the two predominant methods of cooking, in rural Bagamoyo 57% of participants cooked with charcoal, and 33% with firewood, whereas in urban Mwananyamala, 69% cooked with charcoal and only 2% with firewood. In the urban setting, twice as many participants cooked with gas (12% vs 6%) as compared to the rural setting.

Overall, 38% of participants reported some degree of shortness of breath during daily activities. 26% felt limited in their daily activities or at work by shortness of breath (for severity gradings see Table 2). 20% of participants reported at least occasional coughing, and 8% of participants reported that they were previously given a diagnosis of asthma by a healthcare provider (in this study setting, a diagnosis of asthma is usually not supported by spirometry). Interestingly, 47% of participants had a body mass index (BMI) of ≥ 25 and were classified as either pre-overweight or overweight in body mass index.

**Table 2:** Respiratory symptoms, ability to work and body mass index in *N* = 997 participants

Symptom / functional limitation	Severity	% of participants (*n* = 997)
**Shortness of breath**	None	62%
	light	26%
	moderate	10%
	severe	2%
**Limitations due to shortness of breath at home or at work**	no limitation	74%
	slight limitation	16%
	moderate limitation	7%
	severe limitation	3%
**Refrained from taking a job due to shortness of breath**	No	84%
	Yes	16%
**Cough**	I never cough	80%
	I cough occasionally, but not every day	15%
	I cough occasionally, but almost every day	3%
	I cough on almost every day	2%
	I suffer from coughing all the time	0%
**Self-reported previous diagnosis of “asthma”**	Yes	8%
	No	92%
**Weight**	Underweight (BMI < 18.5)	7%
	Normal (BMI 18.5–24.9)	44%
	Pre-overweight (BMI 25–29.9)	32%
	Overweight (BMI ≥ 30)	15%
	No data	1%

### Risk factors for increased SpCO

**Fig. 2:**
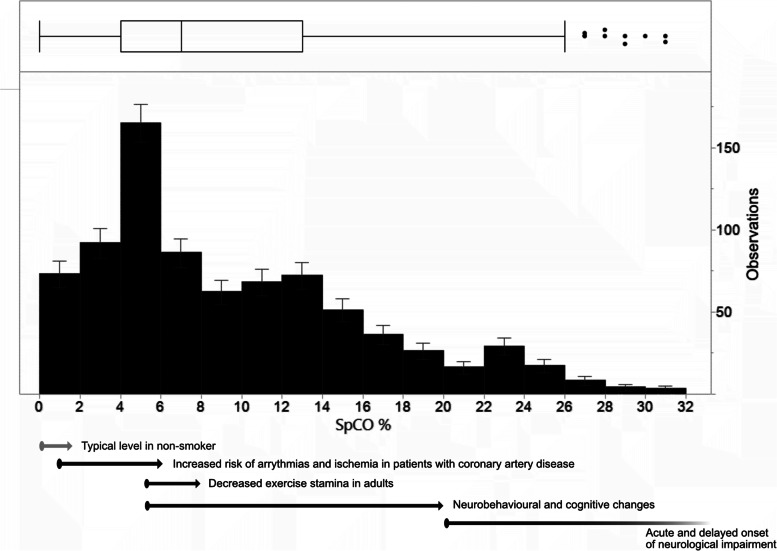
Distribution of SpCO in the study population without active smoking status (*N* = 808). Box and whisker diagram: whiskers indicate outermost data point falling into 1^st^ quartile -1.5 × interquartile range or 3^rd^ quartile + 1.5 × interquartile range. Ranges for adverse effects are indicative only and were obtained from data from the U.S. Dept. of Health and Human Services, Agency for Toxic Substances and Disease Registry [14]

Smoking, but also non-smoking participants had high levels of SpCO: participants who were active smokers had a median SpCO level of 13% (IQR 8–20), whereas participants who were never- or ex- smokers had SpCO levels of 7% (IQR 4–13) and 8% (IQR 4–13), respectively (*p* < 0.01). The following analyses were therefore carried out only in participants without active smoking status and with a valid reported SpCO reading (*N* = 808). The median level of SpCO in the study population without active smoking status was 7% (IQR 4–13, range 2–31%), and the mean level was 9.08% (± SD 6.74), see Fig. 2. 159 participants (19.7%) had an SpCO level of ≥ 15% and 77 (9.5%) of ≥ 20%.

Results of univariate analysis are presented in Table 3. There was no difference between men and women in terms of SpCO (7% in both groups, *p* = 0.76). Likewise, there was a similar level of SpCO across all 7 age groups ranging between median levels of 5% and 9% without significant differences (*p* = 0.59). There was no difference in SpCO between patients and visitors to the hospital (both median SpCO of 7%, *p* = 0.84). Interestingly, participants from the urban site Mwananyamala had higher SpCO levels than participants from rural Bagamoyo (SpCO 10% vs. 6%, *p* < 0.01).

**Table 3:** Univariate analysis of risk factors for increased SpCO (upper section) and symptoms / limitations (lower section) in participants without active smoking status in (*N* = 808 participants). * Only three participants cooked with electricity

		**n**	**Median** SpCO **(%)**	**IQR**	**p** (comparisons between sub-groups, only shown when *p* < 0.1)	**p** (all groups compared)
**Risk factors for increased SpCO values**						
**Age**	18–29	155	7	4–12		0.59
	30–39	121	7	4.5–12		
	40–49	221	8	4–14		
	50–59	142	7.5	4–13		
	60–69	98	9	4–15		
	70–79	60	6	2.25–13		
	≥ 80	11	5	1–11		
**Sex**	Male	349	7	4–12		0.76
	Female	459	7	4–14		
**Type of participant**	Patient of outpatient department	315	7	4–13		0.84
	Accompanying person or visitor	493	7	4–13		
**Area of residence**	Urban	230	10	5–14		< 0.01
	Rural	578	6	4–12		
**Passive exposure to cigarette smoking**	No	387	6	4–13		0.06
	Yes	421	8	4–13		
**Place of cooking in household** (no data *n* = 24)	Outside	321	9	4–14	< 0.01: outside vs. inside – separate kitchen < 0.01: inside – living room vs. inside – separate kitchen	< 0.01
	Inside – living room	110	10.5	5–15		
	Inside – separate kitchen	353	6	4–12		
**Cooking activity** (no data *n* = 3**)**	Regular cooking	500	7	4–14		0.63
	No regular cooking	305	7	4–12		
**Cooking fuel / method of cooking** (no data *n* = 23)	Wood on open fire	178	8	3.75–13	< 0.01: wood in stove vs. gas/electricity 0.07: charcoal vs. gas/electricity	0.1
	Wood in stove	14	11.5	7–13		
	Charcoal	511	7	4–14		
	Gas/electricity	63*	5	3–11		
	Other	19	9	5–16		
**Biomass vs. non-biomass cooking fuel** (no data *n* = 23)	Wood/charcoal	703	7	4–13	0.06: gas/electricity vs. other 0.06: wood/charcoal vs. gas/electricity	0.1
	Gas/electricity	63*	5	3–11		
	Other	19	9	5–16		
**Occupational exposure to dusts vs. fumes**	No exposure	161	6	4–12	< 0.01: dust exposure vs. Mixed exposure: 0.05: dust exposure vs. Fume exposure	< 0.01
	Dust exposure	257	5	4–12		
	Fume exposure	8	10	5.75–18.5		
	Mixed exposure	382	9	4–14		
**Symptoms and limitations according to SpCO values**						
**Severity of shortness of breath**	0 (none)	499	6	4–12	0.02: severity 0 vs. severity 2	0.11
	1 (light)	201	8	4–14		
	2 (moderate)	89	9	4–16		
	3 (severe)	19	7	3–15		
**Limitations due to shortness of breath at home or at work**	0 (none)	599	7	4–12		0.26
	1 (light)	131	8	3–13		
	2 (moderate)	57	9	4–16		
	3 (severe)	21	11	4.5–15		
**Refrained from taking a job due to shortness of breath**	No	686	7	4–12		< 0.01
	Yes	122	10	4–15.25		

Exposure to cigarette smoke contributes to chronic carbon monoxide poisoning; participants exposed passively to cigarette smoke (self-reported) tended to have a higher median SpCO level of 8% as compared to those who were not (median 6%, *p* = 0.06).

The method of cooking and the cooking fuel showed relevant differences in SpCO. Participants cooking with gas or electricity had the lowest SpCO levels (median 5%), followed by participants cooking with charcoal (median 7%). Cooking with wood, particularly using a stove, resulted in highest SpCO (median 11.5%). A combined analysis comparing the biomass fuels wood and charcoal with gas or electricity showed a lower median SpCO of 5% for the latter participants (*p* = 0.06).

The place where cooking takes place seems to be relevant for CO exposure. When cooking takes place in a separate room in the household, participants had the lowest SpCO levels in the blood (median 6%), whereas no difference was observed between outside cooking and cooking in a shared living room (9% vs. 10.5%, *p* = 0.28). In line with the generally lower SpCO values in Bagamoyo, cooking in a separate room was more frequently found in rural Bagamoyo (33.7%) than in urban Mwananyamala (9.2%). Interestingly, there was no difference in SpCO between participants who cooked regularly and those who did not (both 7%, *p* = 0.63).

Participants who reported occupational exposure to fumes (e.g. as a cook, or in a factory) vs. dusts alone had higher SpCO (median 10% for fume or 9% for mixed exposure), as compared to dust exposure alone (median SpCO 5%). In multivariate analysis, cooking in a kitchen separated from other rooms in the house (as compared to cooking outside), living in a rural setting (as compared to an urban setting) and using non-biomass cooking fuel were the strongest factors associated with a lower SpCO (Table 4) confirming the main results from univariate analysis.

**Table 4:** Multivariate linear regression analysis for risk factors of increased SpCO (dependent variable) in participants without active smoking status (*N* = 808). The analysis was performed including the independent variables with a significance of *p* ≤ 0.1 from Table 3 (using the same nominal values for variables) as in univariate analysis: place of residence (urban/rural), passive exposure to cigarette smoking, place of cooking in household (cooking outside as reference category), cooking fuel / method of cooking (cooking with gas/electricity as reference category), biomass vs. non-biomass cooking fuel, occupational exposure to dusts vs. fumes (no exposure as reference category). A model was fitted using stepwise variable selection. The final model included the variables listed in the table

Variable	Regression coefficient	Standardized regression coefficient	*p*	CI_95_ _lower limit_	CI_95_ _upper limit_
Cooking inside – separate kitchen (vs. cooking outside)	-0.29	-0.12	< 0.01	-0.46	-0.13
Rural setting (vs. urban)	-0.3	-0.12	< 0.01	-0.5	-0.13
Cooking with biomass fuels (vs. gas/electricity)	0.26	0.07	0.04	0.02	0.5

### Symptoms and clinical findings associated with increased SpCO

With regard to impact of chronic CO-poisoning on daily life and occupation, there were increasing SpCO levels from participants who had no shortness of breath (6%) towards the two next and higher levels of shortness of breath (8% and 9%, respectively). There was no significant difference for comparison of all severity groups (*p* = 0.26); the comparison of no shortness of breath vs. moderate shortness of breath (*p* = 0.02) showed however a significant difference. A similar increase was seen when participants were asked to grade their physical limitations at home or at work due to shortness of breath (7% ("none") increasing to 11% ("severe"), not significant for comparison of all groups, *p* = 0.26). Eventually, there was a significant difference in SpCO between those participants who had previously refrained from taking a job due to shortness of breath (median 10% SpCO) vs. those who had not (median 7% SpCO, p < 0.01). In multivariate analysis however, this factor was more associated with a rural vs. an urban place of residence and cooking with wood on open fire (vs. cooking with gas/electricity), and less with SpCO (for details see Table 5).

**Table 5:** Logistic regression analysis analyzing influence of SpCO and other variables on participants self-reporting to have "refrained from taking a job due to shortness of breath" (dependent variable) in participants without active smoking status (*N* = 808). Independent variables included age and sex, SpCO and other variables when p was ≤ 0.1 from Table 3 (using the same nominal values for variables) as in univariate analysis: place of residence (urban/rural), passive exposure to cigarette smoking, place of cooking in household (cooking outside as reference category), cooking fuel / method of cooking (cooking with gas/electricity as reference category), biomass vs. non-biomass cooking fuel, occupational exposure to dusts vs. fumes (no exposure as reference category). The final model was adapted using forward selection of variables and included the variables listed in the table

**Variable**		**OR ± CI**_**95**_	***p***
SpCO		1.05 (1.02–1.08)	< 0.01
Rural setting (vs. urban)		1.86 (1.05–3.36)	0.04
Method of cooking	Gas / electricity	Reference category	
	Wood on open fire	4.62 (1.55–13.72)	0.01
	Wood in stove	2.12 (0.35–13.17)	0.41
	Charcoal	1.68 (0.59–4.85)	0.33
	Other	< 0.01	1.0

For participants with valid SpCO readings, additional data on lung function were available from the dataset of TLHS in *N* = 589 participants. Severity of pulmonary obstruction as determined by the Tiffeneau index FEV1/FVC post bronchodilator did not correlate significantly with SpCO (r^2^ = 0.0031, *p *= 0.17) in univariate analysis. When controlled for age as the physiologically most important factor influencing the FEV1/FVC ratio, a significant correlation was observed; SpCO had however no clinically relevant influence on the Tiffeneau index as compared to the influence of age (for details see Table 6).

**Table 6:** Multivariate linear regression analysis for risk factors of pulmonary obstruction as determined by FEV1/FVC ratio post bronchodilator (dependent variable) in participants without active smoking status and with available and valid spirometry data (*N* = 589). FEV1/FVC is decreasing physiologically with age; this variable and also sex were included into the analysis. A model was fitted using stepwise variable selection. The final model included the variables listed in the table

Variable	Regression coefficient	Standardized regression coefficient	*p*	CI_95_ _lower limit_	CI_95_ _upper limit_
Age	-0.001	-0.29	< 0.01	-0.001	-0.001
SpCO	< 0.001	-0.08	0.05	-0.001	< 0.001

## Discussion

This study demonstrated a high burden of symptoms and functional limitations resulting from respiratory causes as well as a high level of CO poisoning in an urban as well as rural setting in Tanzania. Like in many low- to middle-income countries, there is a high and underestimated burden of chronic lung disease in sub-Saharan Africa [19]. Respiratory symptoms occur frequently in daily life, whereas knowledge about causative factors and underlying medical conditions is poor [20]. Respiratory symptoms not only impair quality of life, but incur relevant healthcare seeking and medical care costs: coughing with blood, wheezing and breathlessness cause costs of around 7 US$ whereas simple coughing causes around 3.6 US$ in a 12-month period in rural Malawi [21]. More than one third of participants reported some degree of shortness of breath in daily life in this study, 10% reported limitation of daily activities, and 16% had previously declined a job due to shortness of breath. In another large study from rural Tanzania close to an area with mining industry, 44.5% of participants reported coughing and 7.4% reported shortness of breath as well as limitations of activities of daily life or work absenteeism due to respiratory symptoms [5]. Together, these figures illustrate the high proportion of the local Tanzanian population suffering from respiratory symptoms resulting in limitations of everyday life. Respiratory symptoms also were previously reported to be associated with exhaled CO in a study on household air pollution in Guatemala [22].

Previous studies on chronic CO poisoning in African and other settings determined the severity of CO poisoning by measuring exposure and determining CO levels in the ambient air of houses rather than measuring actual CO poisoning in individual people [5, 16–18]. This method is technically more complex and is less suited to determine person-individual risk factors and symptoms/limitations associated with it. A study in Malawi among children under 5 years of age showed that even individual personalized ambient air CO measurements correlated poorly with CO-Hb in the blood [12]. Non-invasive measurement of SpCO in the blood of individual exposed subjects however allows to determine a more accurate individual profile of actual CO poisoning instead of measuring exposure only. With a half-life of CO-Hb of 320 min. in the blood [23], this method is also less prone to rapidly changing variations of CO in the air (e.g. by rapidly changing room ventilation).

The manufacturer of the Masimo Rad-57 device gives an accuracy of ± 3% for SpCO readings [24]. The device has been clinically validated in several studies, mostly in emergency room settings: a large study found that 73% of SpCO measurements fell within ± 3% of CO-Hb measurements in the blood, and 95% of measurements within 6 percentage points range [25]. This study also reported a rate of 9% of cases with deviation of ≥ 3% above the CO-Hb value and a rate of 18% of cases where SpCO was ≥ 3% below the CO-Hb value [25]. Other studies reported very different observations according to individual pre-specified outcome thresholds for accuracy, sensitivity and specificity with regard to screening individual patients in a triage situation [26–29]. It can be concluded from this data that the device is less suited for patient-individual screening and decision making in triage situations, but accurate enough in the majority of patients to detect and compare exposure levels across different risk groups, typically smokers vs. non-smokers [27]. This assumption is confirmed by the data from this study in Tanzania, where Masimo Rad-57 detected the expected relevant differences in SpCO between active smokers vs. non-active smokers (13% vs. 7% SpCO, p < 0.01) in this study population, confirming the general validity of this method for population based research and risk factor analysis in our setting.

The data show unexpected high levels SpCO in the non-smoking study population (mean 9.8% SpCO) which is far above the normal expected value of < 2% [13]. To date, only one study from sub-Saharan Africa reported SpCO values using the same methods in a paediatric population in Malawi with a mean of 5.8% [12]. Events of chronic CO poisoning have been shown to be associated with dementia [30], major cardiovascular events [31, 32], ischemic stroke [33] and diabetes [34] and also increased blood pressure in pregnant wormen [17]. Our observations therefore raise concerns about a substantial contribution of chronic CO-poisoning to the burden of chronic morbidity in the Tanzanian population and justify further investigations on a larger scale.

A previous study in Tanzania demonstrated levels of CO in the ambient air above the WHO defined acceptable threshold in 38% of rural households during cooking hours [5]. Our data obtained from individually exposed people rather than from ambient air measurements of CO show that a shared exposure caused by the housing situation, the fuel and method of cooking used in a family likely may be a more relevant factor than person-individual risk factors like age group, sex and even cooking activity. Likewise, a kitchen separated from the living room contributed to reducing levels of chronic CO poisoning more than not cooking regularly. The different methods of measuring chronic CO poisoning may explain the difference to other studies which found a sex and age difference by measurement of CO concentration in ambient air [35].

Using charcoal as a cooking fuel is usually associated with the highest indoor air concentration of CO [36]. Our data from individual SpCO measurements did not support this finding and might indicate again that ambient air measurements are less suited to make inferences about individual CO-Hb levels and to determine the risk for secondary complications of chronic CO poisoning. Interestingly, we did not expect to observe a higher level of SpCO in inhabitants of urban vs. rural areas as demonstrated here; cooking in a separate room as a relevant protective factor was however found more frequently in rural Bagamoyo than in urban Mwananyamala where narrower and more crowded living conditions must be assumed. This also underlines the hypothesis of the housing situation being more relevant for CO poisoning than personal individual risk factors. Regarding the relevance of socioeconomic impact of increased SpCO (i.e. having refrained from taking a job due to shortness of breath), uni- and multivariate analyses showed conflicting results. The latter analysis indicates however that direct exposure to particulate matter from cooking with firewood and sociodemographic factors (e.g. more intense physical work in rural vs. urban settings) may predominate as risk factors for this outcome, and it is less dependent on SpCO levels.

This study had limitations. The study was carried out for logistical reasons among randomly selected visitors and patients of an urban as well as a rural health facilitiy. Despite the fact that no differences between visitors and patients were found during the analysis and results seem to be very plausible across all different age groups included, the study formally is not a population representative study. We were not able to directly correlate SpCO measurement results with CO-Hb determined in a blood gas analyzer; typical and expected patterns of chronic CO poisoning among groups of the study population (particularly smokers vs. non-smokers, including the question of passive smoking) were however discernible as expected. This also applies to the fact that the study captured SpCO levels only at one point of time (carbon monoxide has a half-life of 320 min. under room air conditions [13]; correlations found with key variables (e.g. smoking) and chronic symptoms suggest however that also single measurements are indicative of chronic CO poisoning in this setting. In future studies longitudinal measurements will be useful to reduce the influence of day-to-day variability in the study population.

## Conclusion

In conclusion, the data show unexpected high levels of SpCO indicative of chronic CO poisoning in study participants. A substantial burden of disease and a relevant socioeconomic impact caused by respiratory symptoms and CO exposure in people living in urban and rural areas in Tanzania must be assumed. Although non-invasive SpCO measurements may not be suitable to make patient individual treatment decisions, the method is suited to determine CO-Hb levels in different larger population groups and allows to identify typical risk factors where blood gas analyzers are not available and/or non-invasive techniques must be used. The local type of housing shared by all family members and the type of fuel used for cooking are probably more important for reducing personal risk for sequelae of chronic CO poisoning than individual risk factors such as age, sex or specific daily activities among all family members. The findings of this study should be confirmed in large population-representative cohort studies.

## Supplementary Information

**Additional file 1.**

## Data Availability

The datasets used and/or analysed during the current study are available from the corresponding author on reasonable request.
