# Supplementary material for: Symptoms and functional limitations related to respiratory health and carbon monoxide poisoning in Tanzania: a cross sectional study
Source: Environ Health. 2022 Apr 2;21:38. doi: 10.1186/s12940-022-00847-x (PMC8976359; doi:10.1186/s12940-022-00847-x)
Supplement: Supplementary file 1 — Additional file 1. [file 12940_2022_847_MOESM1_ESM.docx]

Supplementary material (online appendix)

| **CRF (as template for eCRF)** | | | | | |
| --- | --- | --- | --- | --- | --- |
| **Personal data *and screening*** | | | | | *Key* |
|  | Patient ID | | \|___\|___\|___\|___\|___\|-\|_3_\|_9_\|-\|___\|___\|___\| | | *1. Study site code (e.g. MWA)*  *2. Subject number (fill empty spaces from left with 0)* |
|  | Date of interview | | \|___\|___\|-\|___\|___\|-\|___\|___\|___\|___\| | | *DD/MM/YYYY* |
|  | Day/Month/Year of birth | | \|___\|___\|-\|___\|___\|-\|___\|___\|___\|___\| | | *DD-MM-YYYY* |
|  | Is day and month of birth known?  Day and Month of birth known | | \|___\| | | *0 = No*  *1 = Yes* |
|  | Sex | | \|___\| | | *0 = Male*  *1 = Female* |
|  | Are you a patient presenting at the hospital or an accompanying person/visitor?Type of study participant | | \|___\| | | *0 = Patient of hospital or clinic*  *1 = Visitor or accompanying person* |
| **Inclusion and exclusion criteria*, informed consent*** | | | | | |
|  | Does subject meet inclusion criteria for any of the two study populations? | | \|___\| | | *0 = Subject does not meet inclusion criteria*  *1 = NTBP: ≥18 years of age; presenting to a primary healthcare facility for any reason other than active pulmonary tuberculosis; no medical history of active TB or prior TB treatment.*  *2 = TBP: ≥18 years of age; medical history of one or multiple previous episodes of active, smear-positive tuberculosis and a minimum time of 9 months after first diagnosis and the day of study inclusion clinical and microbiological (sputum conversion) response to TB treatment at the time of study inclusion; successfully completed TB treatment course at the time of study inclusion; residence in the same catchment area as the NTBP population.* |
|  | 1. Does the patient meet any exclusion criteria? | |  | | 1. Have you been hospitalized for heart disease during the last three months? 2. Have you had a new heart problem or acute heart disease during the last three months? 3. Do you suffer from arterial hypertension without adequate treatment or treatment response? 4. Do you suffer from diabetes without adequate treatment? 5. Are you pregnant? 6. Do you have signs of **acute** infection or fever? 7. Do you have **acute** respiratory problems (e.g., severe coughing, acute shortness of breath)? 8. Are you in acute pain at the moment? 9. Did you undergo thoracic or abdominal surgery during the last three months? 10. Did you smoke during the last two hours? 11. Do you suffer from any other severe physical or mental disease limiting your ability to comply with instructions given or limiting your physical capability for obtaining a valid spirometry result? 12. Does the participant have a resting heart rate of over 100pbm? |
|  | Informed consent | | Date | | DD-MM-YYYY |
|  |  | | Time | | HH:MM |
|  | Informed consent taken before study procedures begin | |  | | Yes/No |
| **Pulse oxymetry and carboxyhemoglobin** | | | | | |
|  | Heart rate | | \|___\|___\|___\| | | bpm |
|  | SpO_2_ | | \|___\|___\|___\| | | % |
|  | SpCO | | \|___\|___\|.\|___\| | | % |
|  | Perfusion Index | | \|___\|___\|.\|___\| | | % |
| **Demographic and social data** | | | | | |
|  | What is the highest level of education you achieved in your life? | | \|___\| | | *0 = No data*  *1 = No education*  *2 = Primary school*  *3 = Secondary school*  *4 = Vocational training*  *5 = College*  *6 = University* |
|  | What is your occupation? | | \|___\| | | *0 = no data*  *1 = farmer/agriculture*  *2 = teacher/education/children*  *3 = business and trade*  *4 = health / medical*  *5 = household work /cleaning*  *6 = driver / transport*  *7 = administrative work*  *8 = seamen / fishing*  *9 = construction work*  *10 = security / police*  *11 = craftsmen*  *12 = cement factory*  *13 = students in education (school, college, university, other training)*  *14 = other*  *15 = not working* |
|  | How many rooms are available in your household?for | | \|___\| | | 0 = no data  1 = one room  2 = two rooms  3 = three rooms  4 = four rooms  5 = five and more rooms |
|  | How many persons live in your household ? | | \|___\| | | 0 = no data  1 – 99 |
|  | What is the leading reason for you seeking medical attention? | | \|___\|  if value = 9, please specify:  \|_____________________________\| | | *0 = no data*  *1 = ENT*  *2 = Eye problems*  *3 = Cardiovascular*  *4 = Lung / breathing*  *5 = Gastrointestinal*  *6 = Urological / reproductive system*  *7 = Musculosceletal / injury*  *8 = Infection / fever*  *9 = Other* |
|  | Are you or have you ever been a smoker? | | \|___\| | | *0 = no data*  *1 = has never smoked before*  *2 = current or recent smoker*  *3 = has smoked previously, but has refrained from smoking at least for the past six months* |
|  | Have you smoked within the last 24 hours? | | \|___\| | | *0 = no data*  *1 = subject did smoke within the last 24 hours*  *2 = subject did not smoke within the last 24 hours* |
|  | How many packs of cigarettes have you smoked during how many years? | | *\|___\|___\|___\|* | | *Number of PackYears smoked in life* |
|  | Are you exposed to cigarette smoke at home or at work (passive smoking)? | | \|___\| | |  |
|  | Are you or have you ever been a user of inhalable substances (e.g. drugs)? | | \|___\| | | *0 = no data*  *1 = does not inhale substances/drugs and has never done so before*  *2 = current or recent user of inhalable substances/drugs*  *3 = has used inhalable substances/drugs previously, but has refrained from doing so at least for the past six months* |
|  | For how many years did you use inhalable substances? | | *\|___\|___\|* | | *Number of years used inhalable substance* |
|  | Are you exposed to inhalable dusts and fumes at your workplace? | | \|___\| | | *0 = no dust or fume exposure*  *1 = exposure to dusts*  *2 = exposure to fumes*  *3 = exposure to multiple types (dusts and/or fumes and/or others)* |
|  |  | |  | |  |
|  | Place of residence | | \|___\| *Reference point # on map: done* | | *Reference point # on map: done* \|___\| |
|  |  | |  | |  |
| **Exposure to inhalable noxious substances** | | | | | |
|  | **Predominant situation during past three years:** | |  | |  |
|  | Do you cook for yourself and/or others on a regular basis?Cooking (refers to study participant only) | | \|___\| | | *1 = regular cooking for yourself or family/friends*  *2 = regular cooking as occupation*  *3 = regular cooking both for family and as occupation*  *4 = no regular preparation of meals* |
|  | What is the predominant method of cooking in your household?  Method of cooking at home | | *\|___\|* | | *0 = no data*  *1 = wood in open fire*  *2 = wood in stove*  *3 = charcoal*  *4 = gas*  *5 = electricity*  *6 = other* |
|  | What is the setting of cooking in your household?  Spatial setting of cooking | | *\|___\|* | | *0 = no data*  *1 = cooking outside*  *2 = cooking inside, cooking in shared room for living AND sleeping*  *3 = cooking inside, cooking place in living room, separate from sleeping room*  *4 = cooking in separate room (kitchen)* |
|  | **In childhood (≤14 years of age)** | |  | |  |
|  | What was the predominant method of cooking in your household during your childhood (≤14years of age)? | | *\|___\|* | | *0 = no data*  *1 = wood in open fire*  *2 = wood in stove*  *3 = charcoal*  *4 = gas*  *5 = electricity*  *6 = other* |
|  | What was the setting of cooking in your household during childhood (≤14years of age)? | | *\|___\|* | | *0 = no data*  *1 = cooking outside*  *2 = cooking inside, cooking in shared room for living AND sleeping*  *3 = cooking inside, cooking place in living room, separate from sleeping room*  *4 = cooking in separate room (kitchen)* |
| **Medical history** | | | | |  |
|  | | Do you suffer currently of any of the following medical conditions?  *Exclude:*  *- Childhood infection, if cured- Infections/Fevers >3 months ago if cured*  *- Trauma*  *- Pregnancy and birth related problems* | YES NO  A \|Hypertension___________\| O O  B \|Heart failure____________\| O O  C \|Ischemic heart disease___\| O O  D \|Diabetes mellitus________\| O O  E \|Asthma_______________\| O O  F \|Cancer________________\| O O  G \|Rheumatic diseases_____\| O O  H \|HIV-infection____________\| O O  I \|________________________\|  J \|________________________\|  L \|________________________\| | | *Yes/No*  *Text* |
|  | | Do you take any of the following medications? | YES NO  Beta-blocker O O  Amiodarone O O  Beta-blocker AND amiodarone\| O O  Any other medication O O | | *Yes/No* |
|  | | Do you take medication to relieve respiratory symptoms ? | YES NO  O O  If no, go to TB-History | | *Yes/No* |
|  | | Do you know the drugs you are taking for respiratory problems ? | YES NO  O O  If no, go to TB history | | *Yes/No* |
|  | | Do you take any of the following respiratory medications?respiratory | YES NO  Short-acting beta-agonist O O  Short-acting anticholinergic O O  Long-acting beta-agonist O O  Long-acting anticholinergic O O  Theophylline O O  Corticosteroids by inhalation O O  Corticosteroids orally O O | | *Yes/No* |
| **TB history** | | | | |  |
|  | **Only for patients in TB-population:** | |  | |  |
|  | | How many episodes of active TB have you had in your life? | *\|___\|* | | *1-9* |
|  | | **FOR EACH EPISODE OF ACTIVE TB:** |  | |  |
|  | | Episode #1  Calendar year of first episode | *\|___\|___\|___\|___\|* | | *YYYY* |
|  | | Episode #1  Duration of treatment | *\|___\|___\|* | | *Months* |
|  | | Episode #2  Calendar year of second episode | *\|___\|___\|___\|___\|* | | *YYYY* |
|  | | Episode #2  Duration of treatment | *\|___\|___\|* | | *Months* |
|  | | Episode #3  Calendar year of third episode | *\|___\|___\|___\|___\|* | | *YYYY* |
|  | | Episode #3  Duration of treatment | *\|___\|___\|* | | *Months* |
|  | |  |  | |  |
| **Lung health assessment** | | | | | |
|  |  | |  | | *Please choose values according to severity of complaints from 0 (no complaints) to 5 (severe complaints)* |
|  | Do you experience shortness of breath when walking up a hill and/or climbing stairs and/or running? | | *\|___\|* | | *0 = no shortness of breath*  *1 = light shortness of breath\*  *2 = moderate shortness of breath*  *3 = severe shortness of breath* |
|  | Do you have the feeling of being limited in your daily activities and/or work by shortness of breath? | | *\|___\|* | | *0 = no limitation*  *1 = slight limitation*  *2 = moderate*  *3 = severe* |
|  | Do you have the feeling of being limited in your activities at home due to shortness of breath? due to shortness of breath | | *\|___\|* | | *0 = no limitation of carrying out activities at home*  *1 = slight limitation of carrying out activities at home*  *2 = moderate limitation of carrying out activities at home*  *3 = severe limitation of carrying out activities at home* |
|  | Do you cough sometimes? | | *\|___\|* | | *0 = I never cough*  *1 = I cough occasionally, but not on every day*  *2 = I cough occasionally, but almost every day*  *3 = I cough regularly on every day*  *4= I suffer from coughing all the time* |
|  | Did you cough with sputum for ≥2 months in two consecutive years? | | *\|___\|* | | *0 = no*  *1 = yes* |
|  | Have you ever refrained from doing an activity or taking a job because of shortness of breath? | | *\|___\|* | | *0 = no 1 = yes* |
|  | Have you ever had an episode of acute bronchitis? | | *\|___\|* | | *0 = never had bronchitis*  *1 = 1 episode of bronchitis*  *2 = 2 episodes of bronchitis*  *3 = 3 episodes of bronchitis*  *4 = 4 episodes of bronchitis*  *5 = 5 and more episodes of bronchitis* |
|  | Have you ever been diagnosed with pneumonia? | | *\|___\|* | | *0 = never had pneumonia*  *1 = 1 episode of pneumonia*  *2 = 2 episodes of pneumonia*  *3 = 3 episodes of pneumonia*  *4 = 4 episodes of pneumonia*  *5 = 5 and more episodes of pneumonia* |
|  | Have you ever been diagnosed with asthma before ? | | *\|___\|* | | *0 = no*  *1 = yes* |
|  | Did you use a health service in the last 12 months for respiratory problems? | | *\|___\|* | | *0 = no health service use*  *1 = 1 health service use*  *2 = 2 health service uses*  *3 = 3 health service uses*  *4 = 4 health service uses*  *5 = 5 or more health service uses* |
|  | Did you use a health service in the last 60 months for respiratory problems? | | *\|___\|* | | *0 = no health service use*  *1 = 1 health service use*  *2 = 2 health service uses*  *3 = 3 health service uses*  *4 = 4 health service uses*  *5 = 5 or more health service uses* |
|  |  | |  | |  |
| **Spirometry** | | | | | |
|  | | Body weight | *\|___\|___\|___\|* | | *kg* |
|  | | Height | *\|___\|___\|___\|* |  |  |
|  | | FEV1 | *\|___\|___\|___\|___\|* | | *ml* |
|  | | FEF25 | *\|___\|___\|, \|___\|___\|* | | *l/s* |
|  | | FEF50 | *\|___\|___\|, \|___\|___\|* | | *l/s* |
|  | | FEF75 | *\|___\|___\|, \|___\|___\|* | | *l/s* |
|  | | FEF25-75 | *\|___\|___\|, \|___\|___\|* | | *l/s* |
|  | | PEF | *\|___\|___\|, \|___\|___\|* | | *l/s* |
|  | | VC | *\|___\|___\|___\|* | | *%* |

Supplementary table 1. Case report form of Tanzanian Lung Health Study as a template for the electronic case report form.
